# A rare endoscopic appearance of granulomatosis with polyangiitis involving the intestine: a case report

**DOI:** 10.1186/s12876-018-0885-9

**Published:** 2018-10-25

**Authors:** Sheng-Wu Pan, Chang Wang, Xin Zhang, Li Zhang, Qi-Qi Yan, Cai-Juan Zhao, Cheng Chang, Xiao-Dong Luan

**Affiliations:** 1 Department of Gastroenterology, People’s Liberation Army 264 Hospital, No. 30, Qiaodong Street, Yingze District, Taiyuan, 030000 Shanxi Province China; 2 Public Relations Branch,Taiyuan Red Cross Blood Center, Taiyuan, 030022 China

**Keywords:** Granulomatosis with polyangiitis, Wegener’s granulomatosis, Gastrointestinal tract bleeding, Hemorrhagic colitis, Intestinal tract

## Abstract

**Background:**

The involvement of granulomatosis with polyangiitis is less frequent in the intestine.

**Case presentation:**

We present a case of Wegener’s granulomatosis with unusual endoscopic appearance, involvement in a young man’s gastrointestinal tract. A 45-year-old man was diagnosed with Wegener’s granulomatosis 11 years ago, and relapsed with abdominal pain and melena. A colonoscopy was performed, and the appearance of mucosal lesions with an unusual annular black membrane was observed. A black ring-shaped membranous tissue adhered to the surface of the colon wall, which could be traversed by an endoscopic forepart.

**Conclusion:**

Biopsy of the black membrane revealed degenerative colonic mucosal tissues, while deep colonic biopsy revealed inflammatory granulation tissues. This has not been reported in previous documents.

## Background

Granulomatosis with polyangiitis (GPA), which is also known as Wegener’s granulomatosis (WG), is a necrotizing granulomatous vasculitis with small arteriovenous and capillary involvement [1]. At present, its cause remains unknown, and it belongs to autoimmune diseases. The clinical manifestation is usually multi-system organ damage, which mainly involve the upper and lower respiratory tract, kidneys and skin. The involvement of the intestinal tract is relatively rare, although several previous articles have reported the clinical manifestations of gastrointestinal tract involvement in GPA [2–7]. However, the endoscopic imaging of intestinal lesions in GPA is relatively rare. Here we report a case of GPA involving the intestine with an unusual endoscopic appearance.

## Case presentation

A 45-year-old male was hospitalized due to bloody nasal discharge, hemoptysis and rash for 11 years, which aggravated after 2 weeks, and presented with abdominal pain and melena for 1 month. The patient was hospitalized and diagnosed with WG 11 years ago. Furthermore, he had rashes on his face, trunk, limbs and feet, and had oral ulcers, perianal ulcers, and sinusitis.

**Fig. 1:**
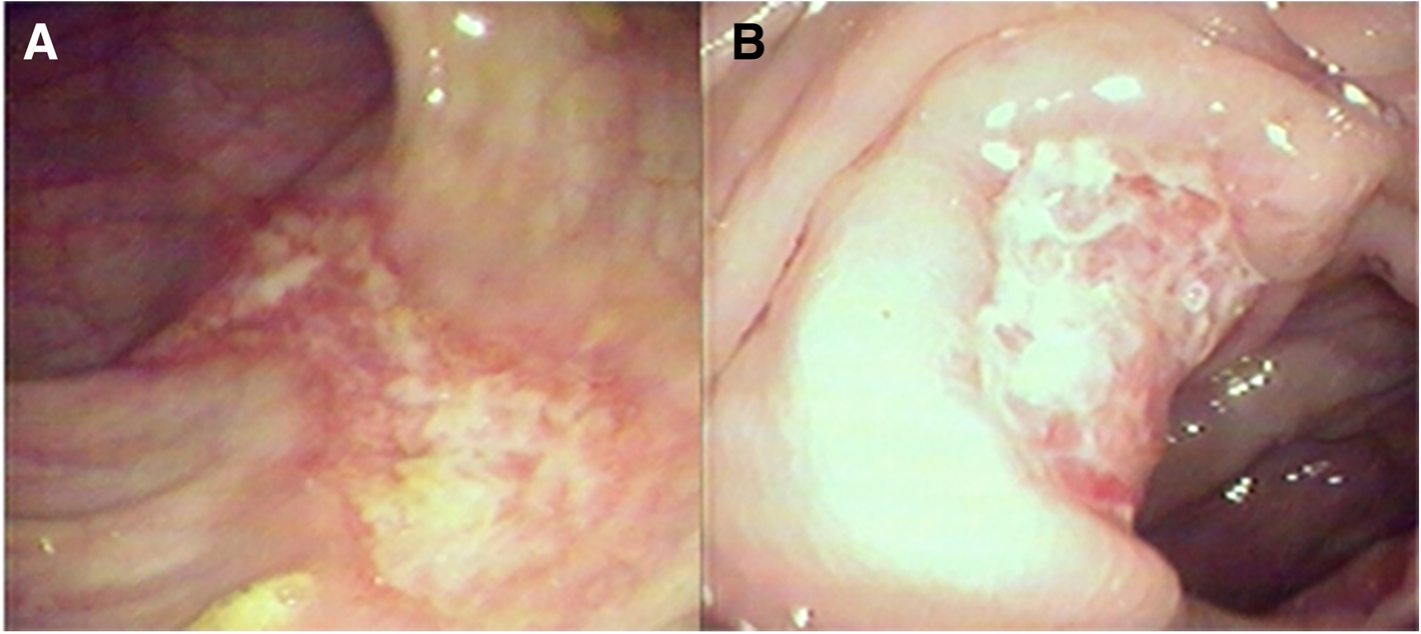
**a** Large ulcer: the base was covered with white fibrin coating. **b** Rectal circular ulcer: the rolled margins are shown

**Fig. 2:**
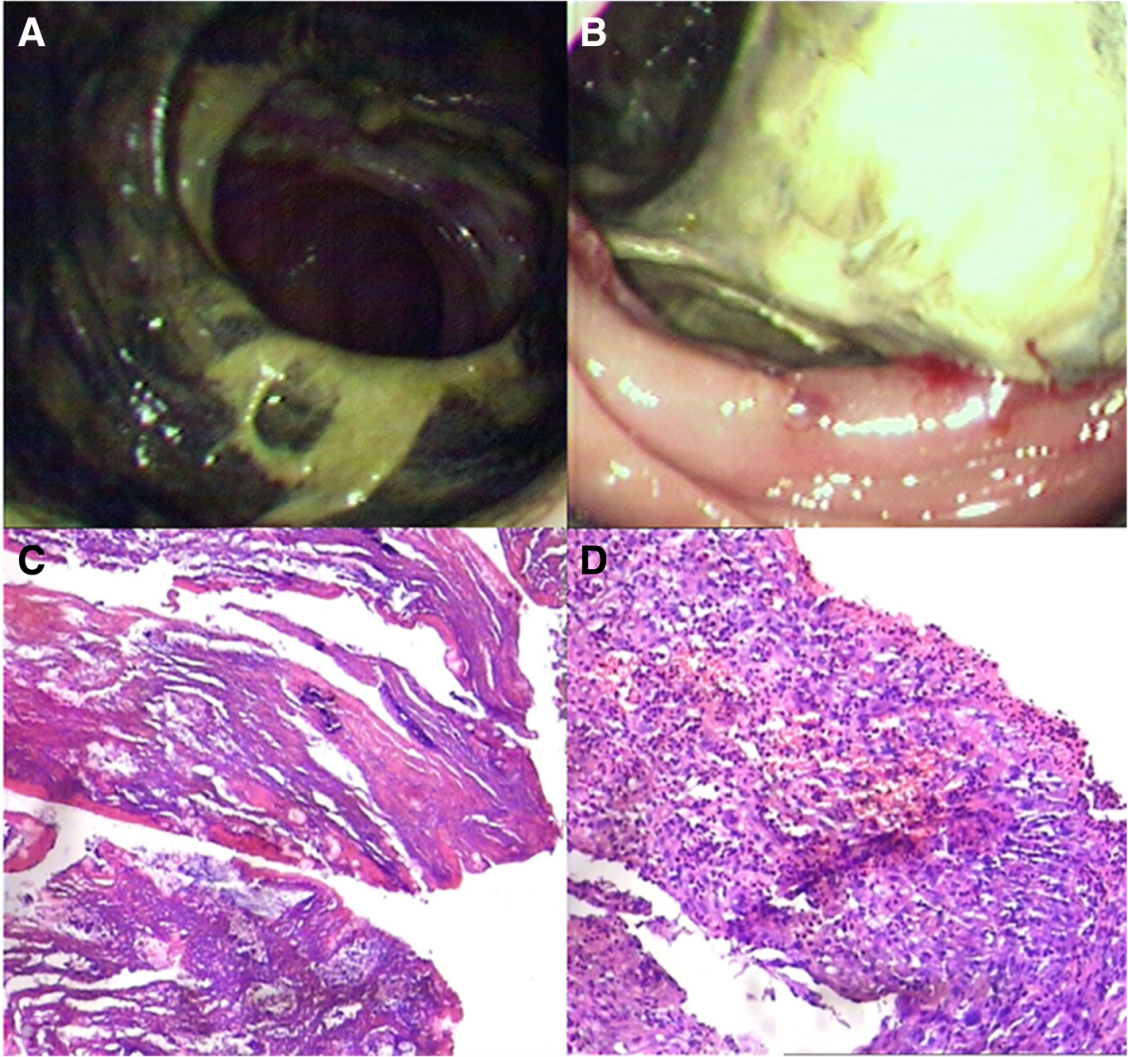
**a** and **b** A black annular membrane, which was tunnel-like, allowing the colonoscopy to pass through. There are scattered erosions and ulcers at the bottom (H&E × 20). **c** Degenerative colonic mucosal tissue: only have contours. **d** Inflammation granulation tissue: no normal colon glands (H&E × 10)

The laboratory tests revealed the following: leucocytes count of 8.5 × 10^9^/L, hemoglobin level of 79 g/L, urine protein (+), microhematuria (+), erythrocyte sedimentation rate of 50 mm/h, c-ANCA (antineutrophil cytoplasmic autoantibodies) with a titre of 1 in 100, and PR3-ANCA (anti-neutrophil cytoplasmic antibodies proteinase 3) of > 200 RU/ml. Computed tomography (CT) revealed left frontal and ethmoid sinusitis, and bilateral maxillary sinusitis. Based on these clinical features and laboratory findings, the patient’s diagnosis of WG was accurate. In order to clarify the cause of the abdominal pain, a colonoscopy was performed with the written informed consent of the patient. Different sizes of irregular ulcerations, which were 3–4 cm and 1–2 cm in diameter, were scattered at different intervals throughout the colon (Fig. 1). The larger ulcers presented raised margins and fibrin coatings on the base. Colonic biopsies revealed inflammatory granulation tissues. At approximately 40 cm from the anus, an annular black membrane, which was approximately 4 cm in diameter, adhered to the surface of the intestinal wall. The membrane could be lifted using biopsy forceps. Part of the membranous substances appeared like mucosal surface tissues, and was completely separated from the intestinal wall. The colonoscope could pass through the membrane, and erosions and ulcers were scattered at the bottom (Fig. 2a and b). The membrane biopsy revealed degenerative colonic mucosal tissues, while the colonic biopsy revealed inflammatory granulation tissues without normal colon glands (Fig. 2c and d). Oral prednisolone (60 mg/d) combined with intravenous cyclophosphamide (600 mg/d, once) was given. The patient was rehabilitated and discharged after 10 days of treatment. Symptoms including bloody nasal discharge, hemoptysis, skin rash and abdominal pain all disappeared.

## Discussion and conclusions

Granulomatous vasculitis is a necrotizing small-vessel vasculitis, and granulomatous inflammation is strongly associated with anti-neutrophil cytoplasmic autoantibodies [8]. The typical clinical manifestation is a triple syndrome, that is, upper respiratory disease, pulmonary inflammation and glomerulonephritis. Gastrointestinal tract involvement is uncommon, and is usually found at autopsy. The types of lesions mainly include submucosal edema, ulcers, hemorrhage, mesenteric ischemia, intestinal obstruction and perforation. The diagnosis of gastrointestinal complications depends largely on these clinical manifestations.

Pagnoux et al. [9] conducted a study on 62 patients with systemic necrotizing vasculitis that involved the gastrointestinal tract, and found that the most common symptoms were abdominal pain (97%), nausea, vomiting, diarrhea, bloody stools, and melena. Furthermore, previous studies have also observed these symptoms [10, 11]. The locations of GPA lesions in the gastrointestinal tract are diverse, and can involve all parts of the intestine. The most common pathological manifestations are ulcer, intestinal necrosis and perforation [2, 3, 10, 11].

Although the present understanding of gastrointestinal lesions in GPA patients has increased, there are few reports on the endoscopic appearance of GPA involving the bowel with accompanying images. In previous years, Robin et al. [12] reported a case of GPA with intestinal lesions mainly characterized by ulcers. However, the tissue biopsy was nonspecific inflammation, and lacked the characteristics of granulomatous vasculitis. Endoscopic biopsy findings are often non-specific inflammation, ulcers, or erosions, and these can rarely be diagnosed as GPA. Camilleri et al. [10] pointed out that this may be correlated to superficial tissue biopsy, because the small and medium blood vessels are located deeper under the mucosa. Therefore, it is also necessary to distinguish between infectious bowel diseases, non-infectious inflammatory bowel diseases and ischemic colitis, such as Crohn’s disease characterized by aphthous ulcers, paving stone-like changes, and segmental lesions and ischemic enteritis characterized by longitudinal map-like ulcers. Besides, side effects of immunosuppressants should also be excluded. In the present case of GPA, the colonoscopy has revealed a manifestation of black ring-shaped lesion not previously reported in any disease. The degenerative intestine mucosal tissue formed a black ring-shaped membranous tissue that could be traversed by an endoscope. The intestinal wall biopsies beneath it had characteristics of inflammatory granulation, with no normal colon glands. This has not been observed in previous studies. However, colonoscopy was not performed after treatment with immunosuppressants and glucocorticoids.

In conclusion, when GPA patients suffer from abdominal pain, diarrhea, bloody stools, or melena, the differential diagnosis should be made while taking into account the possibility of granulomatous vasculitis involving the gastrointestinal tract. Colonoscopy combined with tissue biopsy can help to determine the nature of the disease, and provide an effective method for differential diagnosis.

## Abbreviations

GPA: Granulomatosis with polyangiitis
WG: Wegener’s granulomatosis
